# Attention-Guided EfficientNet-B3 with Grad-CAM Visualization for 22-Class Bone Fracture and Anatomical-Region Classification on the MultiBoneX Dataset

**DOI:** 10.3390/diagnostics16172841

**Published:** 2026-09-03

**Authors:** Irshad Ahmad, Mian Hafeez Ur Rehman, Saleh M. Altowaijri

**Affiliations:** 1 Department of Computer Science, Islamia College Peshawar, Peshawar 25000, Khyber Pakhtunkhwa, Pakistan; hafeez.icup@gmail.com; 2 Department of Information Systems, Faculty of Computing and Information Technology, Northern Border University, Rafha 91911, Saudi Arabia

**Keywords:** bone fracture, multi-class, multi-region, pre-trained CNN, classification

## Abstract

**Background/Objectives**: To achieve effective clinical decision-making from radiographic images, accurate diagnosis of bone fractures is required, but this is difficult due to anatomical variation, subtle fracture appearance, and variable imaging conditions. Existing deep learning studies for fracture detection are predominantly limited to binary classification or single anatomical regions, limiting their real-world clinical utility. This study demonstrates that a single deep learning model can effectively classify multi-region bone fractures by transforming the task into a 22-class classification problem, utilizing the publicly available MultiBoneX dataset. **Methods**: The proposed model utilizes an EfficientNet-B3 convolutional neural network integrated with a Convolutional Block Attention Module (CBAM) to enhance feature representation by focusing on diagnostically significant areas. We used regularized preprocessing, data augmentation, and structured training to support strong model learning and generalization. Model predictions were interpreted using Gradient-weighted Class Activation Mapping (Grad-CAM) to highlight the image regions that were most important for class selection. **Results**: When tested on a held-out test set of 3280 images, the model achieved an overall accuracy of 75.03% (95% CI: 73.57–76.43%), with precision, recall, and F1-score of 77.09% (95% CI: 75.28–78.77%), 72.85% (95% CI: 71.00–74.60%), and 73.45% (95% CI: 71.49–75.15%), respectively. The overall multi-class Matthews Correlation Coefficient (MCC) was 0.7361, providing an additional class-imbalance-aware measure of classification performance. **Conclusions**: These findings demonstrate the feasibility of a unified, multi-class system for diagnosing bone fractures across diverse anatomical sites, providing a scalable foundation for future AI-assisted radiography.

## 1. Introduction

Bone fractures are among the most common injuries in emergency medicine and orthopedic health care, affecting millions of people worldwide each year [1]. Recent reports have indicated that the number of new cases of fractures each year all over the world is estimated to hit 178 million, with a 33 percent rise compared to the number in 1990 and a significant burden on the healthcare systems [2]. These injuries may result from various factors, such as trauma, accidents, sporting activities, and other pathological conditions that weaken bone structure. To plan treatment properly, minimize complications, and restore normal musculoskeletal function, clinicians must detect bone fractures in a timely and accurate manner. Nevertheless, the large number of radiographs, the complexity of bone structure, and differences in fracture patterns make manual interpretation by radiologists and orthopedic specialists difficult and time-consuming. In addition, image quality and observer experience can affect diagnostic accuracy, leading to misinterpretation or delayed diagnosis [3,4,5]. Conventional computer-aided diagnosis (CAD) devices have been proposed to aid clinicians, but they rely on manually designed features, and their limited generalization across body parts has restricted clinical use. Over the past few years, artificial intelligence (AI), especially deep learning methods, has reshaped medical image analysis [5]. Convolutional Neural Networks (CNNs) and their derivatives excel at image recognition tasks by automatically learning hierarchical feature representations directly from data [6]. CNN-based medical imaging models have matched or exceeded expert performance in detecting wrist and hip fractures (as well as the ankle and other bones) [7]. Despite these achievements, most available research is limited to a single bone type or a few classes. It does not provide a more generalizable methodology for addressing fractures across different anatomical locations.

This paper proposes a deep learning model for multi-class bone fracture classification using the publicly available MultiBoneX dataset, which contains radiographic images from 11 anatomical regions, each with fractured and normal images, resulting in 22 unique classes. We used large-scale preprocessing and data augmentation and tested various convolutional neural network (CNN) models. The EfficientNet-B3 [8] model with CBAM [9] achieved the best overall performance. The main objectives of this study are to:

- Explore the possibility of multi-region bone fracture classification on a large scale by posing the task as a 22-class problem;
- Present one of the initial deep learning assessments of the new MultiBoneX dataset;
- Create a single end-to-end classification framework that detects anatomical region and fracture status jointly in one model, for cases where the affected region is not reliably known in advance, such as incomplete imaging-order metadata or trauma presentations spanning multiple candidate regions, etc.
- Avoid the overhead of a hierarchical alternative, which would require training and running up to 11 separate region-specific models, or a two-stage pipeline that classifies the region first and fracture second, by handling both tasks in a single model.
- Find out the performance of attention-based architectures, especially the combination of CBAM and EfficientNet-B3, to enhance the performance of the fracture classifier.
- Use Grad-CAM [10] visualization to identify image regions contributing to the model’s predictions.

Taken together, these objectives highlight a core contribution focused on problem formulation rather than structural model innovation. By framing anatomical localization and fracture classification as a joint prediction task, instead of the multi-stage or hierarchical pipelines used in prior work—which have also typically covered fewer classes—this choice reflects both clinical and practical priorities: handling cases where the body region isn’t known up front, while skipping the hassle of training, deploying, and maintaining multi-stage or site-specific models.

The study will improve clinical assessment, reduce diagnostic variability, and strengthen decision support by integrating this AI-based classification pipeline with radiographic workflows. Finally, this structure is a step towards complete automation and multi-classification of bone fractures, which can further help deploy intelligent diagnostic frameworks in healthcare.

## 2. Related Work

Before the deep-learning era, automated bone-fracture detection largely relied on classical machine-learning (ML) pipelines run on institutionally collected (privatized) radiographic or CT data, as large publicly labeled datasets of fracture images were not available at the time [11,12]. These techniques were usually limited to binary classification (fracture vs. nonfracture) in a single anatomical region (e.g., wrist, tibia, and hip) [13,14]. The general procedure included manual feature extraction, including Gray-Level Co-occurrence Matrix (GLCM), Local Binary Patterns (LBP), Histogram of Oriented Gradients (HOG), wavelet transforms, and classifiers such as Support Vector Machines (SVMs), k-Nearest Neighbors (KNN), Decision Trees (DT), Random Forests (RFs), or Gradient Boosting Machines [15,16,17]. Preprocessing often involved Region of Interest (ROI) segmentation, edge detection, and dimensionality reduction algorithms such as Principal Component Analysis (PCA) and Recursive Feature Elimination (RFE) to optimize the feature set and improve classifier performance [18,19,20].

Several representative ML-only studies exemplify this trend. Bagaria et al. [11] evaluated a wavelet-based segmentation framework paired with machine learning classifiers (SVM and EBP-NN) for bone fracture detection, including long bones such as the tibia. Ahmed [21] compared several standard ML classifiers using texture and morphological features with SVM and RF classifiers to predict the presence of fractures. Park et al. [12] developed a radiomics + SVM pipeline for classifying AO/OTA pelvic fractures, achieving a best Area Under the Curve (AUC) of 0.75. Li et al. [13] examined vertebral-fracture detection using CT radiomics features with SVM and RF classifiers. Saravi et al. [22] combined CT radiomics with clinical data to predict vertebral fracture risk using tree-based ML models and demonstrated the importance of multimodal features in fracture-risk prediction. Collectively, these studies relied on institutionally collected (private) datasets, with several using relatively limited sample sizes, reflecting the data constraints of traditional ML-based fracture analysis.

Other ML pipelines attempted to extend fracture classification beyond single-task detection, moving from binary detection to multi-class fracture classification settings. Several hybrid feature-fusion models combined texture, shape, and wavelet features with ensemble classifiers, including voting and RF/SVM/XGBoost stacking, and reported accuracies ranging from the mid-80s to the low-90s. The literature surveys by Kong et al. [23] and Namireddy et al. [24] emphasize that, despite their effectiveness on specific fracture-detection tasks, classical ML approaches depend heavily on manual feature engineering and relatively homogeneous data. Jung et al. [25] further reported pooled sensitivity and specificity of 92% and 91%, respectively, for AI-based fracture detection using image-based data, while indicating that classical ML methods made a relatively limited contribution to handling complex, heterogeneous fracture-detection tasks. Overall, these studies show that classical ML can perform well for multi-class fracture classification. Still, its reliance on handcrafted features and constrained data limits its ability to generalize across diverse anatomical regions and heterogeneous datasets. These limitations have encouraged the shift toward deep-learning approaches.

Automated bone-fracture classification has increasingly adopted deep-learning techniques, particularly convolutional neural networks (CNNs). Kanagaraj et al. [26] proposed a Node-Level Capsule Graph Neural Network (NCGNN) optimized with the Gazelle Optimization Algorithm (GOA), achieving improved accuracy, precision, and recall compared with state-of-the-art binary deep-learning and machine-learning methods. Uysal et al. [27] developed an ensemble CNN model for shoulder-fracture analysis and achieved 84.7% accuracy on the publicly available MURA dataset, demonstrating the effectiveness of ensemble learning for fracture classification. ElSaadawy et al. [28] proposed a two-stage MobileNet-based approach for seven upper-body bones using the MURA dataset and achieved an average accuracy of 73.42% after combining both classification stages. Dong et al. [29] trained a CNN for osteoporotic compression fracture (OCF) classification using spinal X-rays and reported an AUC-ROC above 0.94 and specificity above 0.99. Similarly, Vellanki et al. [30] proposed a hybrid deep-learning Shuffle Parallel Convolutional Neural Network (ShPCNNet) for shoulder-fracture classification and reported 91.877% accuracy. Other studies have also reported accuracies ranging from 80% to 97% for fracture classification involving the wrist, hip, and hand [31,32,33]. Collectively, these studies demonstrate deep-learning models’ strong capability to learn discriminative fracture-related features. However, most approaches focus on binary classification within a specific anatomical region, rather than providing a unified multi-class framework that covers multiple bone regions and fracture conditions.

Subsequent investigations extended to multi-class fracture classification across a broader anatomical range and fracture-type variations. A multimodal detection system combining X-ray and MRI images to separate 10 fracture types and normal bones, introduced by Parvin and Rahman [34], was trained on a private dataset that reported an mAP50 of 92% and can be inferred in real time. Similarly, Alshahrani et al. [35] trained CNN architectures on the publicly available FracAtlas dataset and achieved 80% accuracy with YOLOv8 and 73% with VGG16. Sarhan et al. [36] and Senanayake et al. [37] used CNN models to classify osteoporosis based on knee and DXA X-rays (private datasets). According to Sarhan et al. [36], classification rates exceeded 0.9, whereas Senanayake et al. [37] reported a mean Area Under the Receiver Operating Characteristic (AUROC) of 0.74 for predicting fracture risk. Although these methods increased the number of classes, most of them still treated each bone region as a separate binary classification task.

More recent studies have further explored hybrid, ensemble, and transformer-based architectures for fracture classification. Lee et al. [38] used Attention U-Net with Inception-ResNet-V2 to achieve 88% accuracy and AUC values ranging from 0.77 to 0.94 on pelvic fracture classification using private data. Medical classification tasks have turned into one of the most successful tasks to apply EfficientNet architectures: an EfficientNet-B6 model in [39] reached 96.83% accuracy on the public FracAtlas dataset, whereas an EfficientNet-B3 ensemble in [40] achieved 83% sensitivity and 81% accuracy on classifying intra- and extra-articular distal radius fractures on clinical X-ray images of the wrist. Researchers have also used vision transformer models for multi-class fracture classification, including a ViT-L/16 that reached 83% accuracy on a seven-class femur dataset [41].

Although these approaches have improved fracture classification, most existing studies focus on individual anatomical regions or a limited number of classes. In contrast, joint multi-region and fracture-status classification has received comparatively little attention.

To address this gap, Barhoom et al. [42] utilized the large-scale public MURA-v1.1 dataset to concurrently classify multiple bone regions and their conditions, achieving a model precision of 85.96%. This line of research was further explored in [43,44] through deep learning-based approaches for multiclass and hierarchical fracture analysis. Phadke et al. [43] employed multiple pretrained CNN architectures for multiclass classification of normal and different fracture types, whereas Madi et al. [44] proposed a three-layer framework that first classifies X-ray images into bone types using a Vision Transformer, followed by bone-specific ResNet50 classifiers for fracture-status prediction and a DETR-based module for fracture localization. Nevertheless, these approaches either focus on direct multiclass fracture-type classification or rely on separate models for anatomical classification, fracture detection, and localization, rather than jointly learning the hierarchical anatomical structure and fracture status within a unified classification framework.

Conversely, the current work presents a deep learning-based multi-region fracture classification framework trained on the publicly available MultiBoneX dataset [45], which comprises 11 anatomical locations with both positive (fractured) and negative (normal) samples for each location. The proposed approach addresses anatomical region recognition and fracture-status classification in a single system by formulating the task as a unified 22-class classification problem—to our knowledge, the largest class count attempted for joint region–fracture classification, compared with 14 classes in the closest prior joint approach [42]. The EfficientNet-B3 architecture, enhanced with Convolutional Block Attention Module (CBAM), achieved an overall test accuracy of 75.03%, demonstrating the feasibility of a unified multi-region fracture classification framework. Unlike prior binary, region-specific, or multistage approaches, this paper provides a generalized multi-region, multi-class model trained on a publicly available dataset, supporting scalable and reproducible AI-assisted bone fracture classification.

## 3. Proposed Method

### 3.1. Dataset

This study’s experimental assessment used the MultiBoneX ver1 dataset [45], an extensive, openly accessible collection of 22,558 high-quality radiographic (X-ray) images created to detect bone fractures and classify them by anatomy. Mogeeb Mosleh (2025) [45] presented and edited it by incorporating images from several open-access repositories: MURA [46], FracAtlas [47], HBFMID [48], and LERA [49]. To make such images clinically reliable and diverse, a multi-stage preprocessing pipeline was used, including data cleaning, verification of annotation by professional radiologists, image normalization and enhancement, and data augmentation.

Each image is hierarchically annotated with two labels: (i) skeletal region (anatomical class), and (ii) Fracture status (positive or negative). This dual-level labeling supports both binary classification (fracture vs. normal) and multi-class classification (based on anatomical region). The dataset encompasses 11 anatomical classes, representing both upper and lower limb structures, as summarized in Table 1.

To facilitate systematic training and assessment of the deep learning model, the dataset was provided with pre-defined training and testing folders, representing 85.5% and 14.5% of the total data, respectively. The training folder was split into training (90%) and validation (10%) using PyTorch’s random_split function, resulting in the following partition:

- Training set: 17,350 images
- Validation set: 1928 images
- Testing set: 3280 images

Neither split was stratified by class, so per-class proportions vary somewhat across subsets. Table 2 presents the per-class distribution of images across the training, validation, and test subsets.

**Figure 1:**
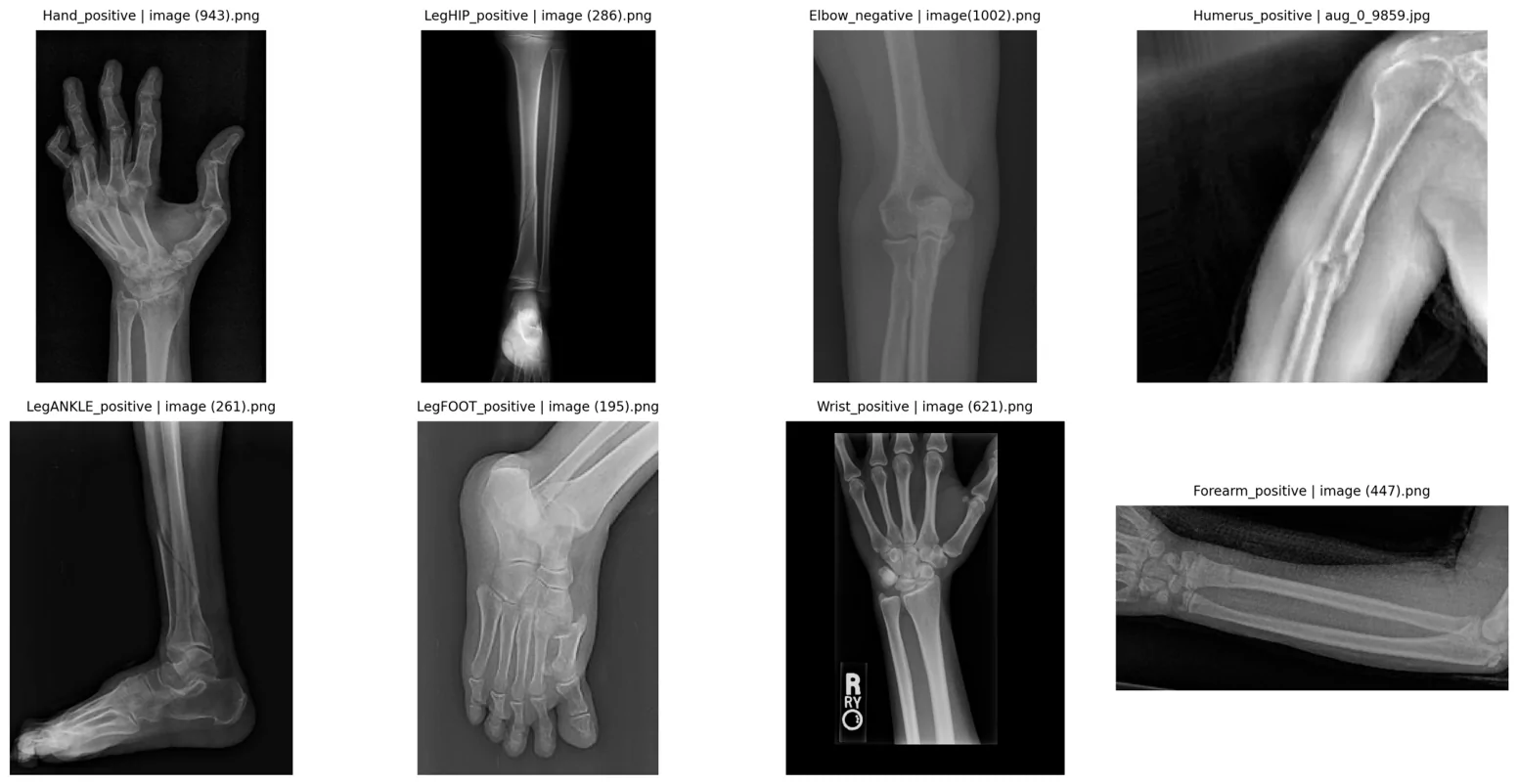
Example Images from the MultiBoneX ver1 Dataset.

This division includes samples of all anatomical classes and fracture types across all subsets, supporting effective model learning. The dataset incorporates images from multiple public radiographic repositories, providing variation in anatomical regions and image acquisition characteristics, and thus offers a solid basis for building and testing deep learning-based computer-aided diagnostic (CAD) systems to classify and detect bone fractures. Some sample images from the MultiBoneX ver1 dataset are shown in Figure 1.

To check for duplicate or near-duplicate images across the data splits, we ran a perceptual-hashing analysis (64-bit pHash) [50] on the full dataset of 22,558 images. We treated images that produced an identical hash as exact duplicates. This turned up 347 duplicate groups, and 178 of these groups had images spread across more than one split. In total, 635 images (2.81% of the dataset) had an exact-hash duplicate sitting in a different split. We also checked for near-duplicates, using a Hamming distance of ≤5, and found 711 cross-split pairs that looked visually near-identical without being hash-identical. This points to a modest but real amount of cross-split leakage, likely a side effect of how MultiBoneX was built: by combining four public source datasets that don’t share a unified deduplication process or patient IDs.

### 3.2. Model Overview

The proposed framework aims to perform multi-region, multi-class bone fracture classification by combining anatomy region recognition and fracture detection into a unified, end-to-end deep network. The objective is a 22-class classification problem. This single formulation provides the capability to learn anatomical context and fracture pattern in a single model, and the need to have many classifiers, each associated with a different bone region, is removed.

Although the anatomical region of a radiograph is typically known at acquisition time from the imaging order, we designed the model to jointly classify region and fracture status for four practical deployment reasons. First, in settings such as emergency triage, resource-limited clinics, or teleradiology queues, structured order metadata is not always reliably attached to the image when the model runs, so a region-agnostic model remains usable even when this metadata is incomplete or missing. Second, trauma presentations frequently do not localize to a single region at the time of ordering; for example, a fall or road traffic accident victim may receive X-rays of several candidate regions (e.g., hip, pelvis, and femur) before it is known which, if any, is fractured. Each resulting image still requires independent region and fracture classification rather than relying on a single assumed region label. Third, a radiograph acquired for one region can incidentally capture adjacent anatomy (e.g., a finger radiograph partially capturing the hand), and a joint model can flag unexpected findings outside the originally ordered region. Fourth, the model’s region prediction can serve as an automated quality-control check, flagging mismatches between the predicted region and the stated order, for example, catching mislabeled or wrong-image-uploaded studies in a PACS pipeline before radiologist review.

The backbone of the proposed system is the EfficientNet-B3 convolutional neural network, chosen for its strong balance of computational efficiency and representational power (Section Computational Comparison and Backbone Selection Justification). CBAM is incorporated into the network to enhance the model’s focus on fracture-relevant regions. This module introduces channel and spatial attention, allowing the network to focus on important regions and ignore irrelevant background information.

**Figure 2:**
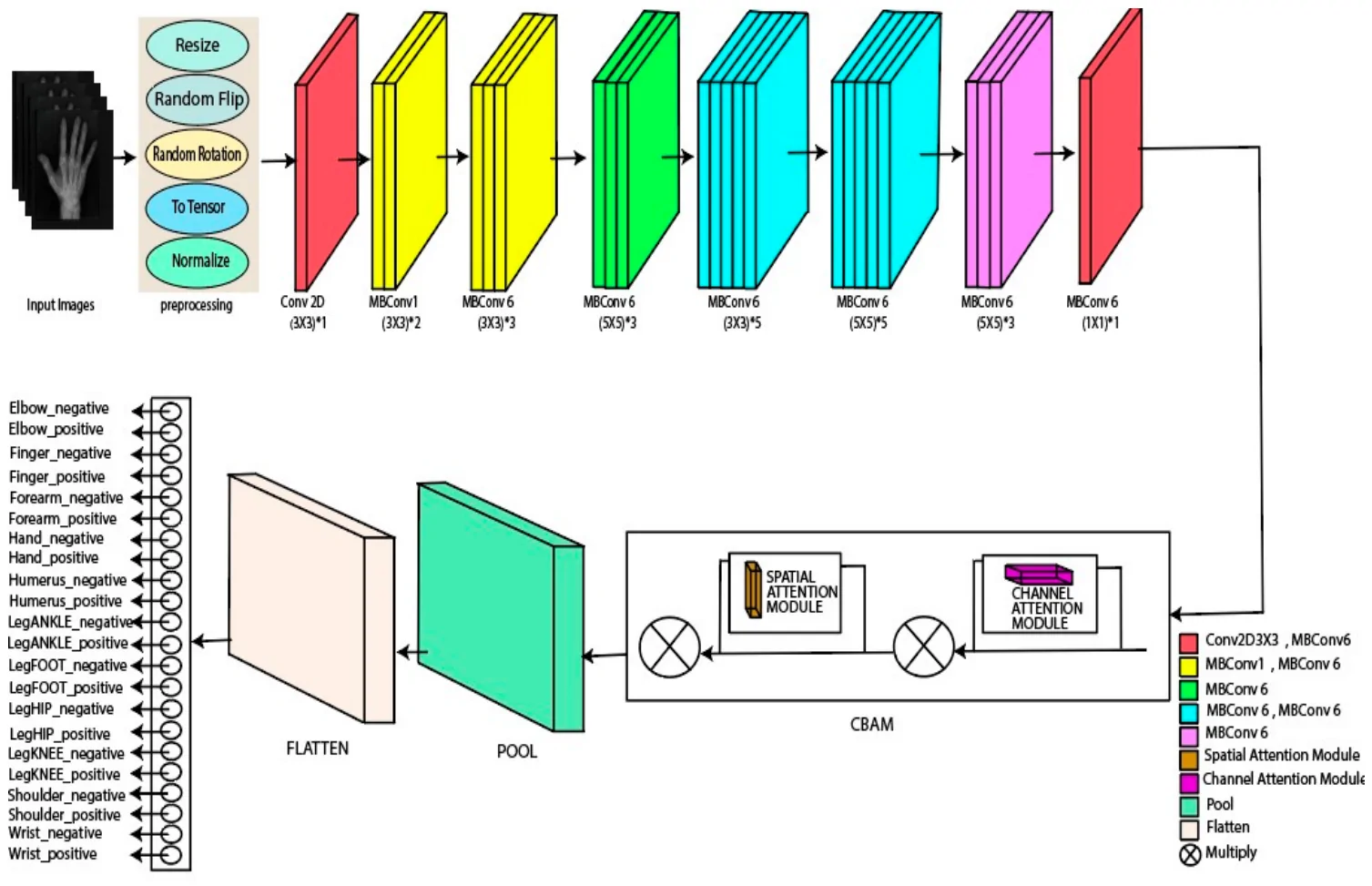
Proposed EfficientNet-B3+CBAM Model Architecture.

Specifically, the CBAM module is inserted after the final MBConv6 block of the EfficientNet-B3 backbone, operating on the resulting feature maps before global average pooling and the classification head, as illustrated in Figure 2.

EfficientNet-B3 is a tuned feature extractor that chooses network depth, width, and resolution to provide high performance at a medium computational cost. CBAM: Focuses the model on informative regions and improves its ability to identify subtle/fine-grained fracture patterns that would have been missed otherwise. EfficientNet-B3 with CBAM allows the model to learn spatial and contextual features for all classes, providing a single and scalable structure that can be used to classify bone fractures in general.

### 3.3. Advantages

The suggested technique has several merits in comparison with the traditional region-specific or binary classification methods:

Unified multi-class learning removes dependence on reliably available region metadata at inference time, supports detection of incidental findings outside the originally ordered region, and enables automated quality-control checks against stated orders, though at the cost of solving a harder joint problem than a region-specific classifier would need to.

Attention-guided feature refinement enhances localization of subtle fracture features.

End-to-end integration simplifies the workflow and eliminates the need for multi-stage pipelines.

Reproducibility and scalability are ensured through the use of publicly available datasets and standardized training protocols.

### 3.4. Statistical Analysis

The statistical evaluation of the proposed model used standard performance metrics for multi-class classification. Overall model performance was assessed using accuracy, precision, recall, F1-score, and Matthews Correlation Coefficient (MCC) computed with the scikit-learn library. We also reported class-wise precision, recall, F1-score, MCC, and support to provide a detailed, per-class assessment across all output classes.

To evaluate model stability and generalization, we performed 3-fold cross-validation on the training dataset, allowing us to compare training and validation performance across folds rather than relying on a single split.

In addition to cross-validation, we computed 95% confidence intervals for the principal test-set metrics using bootstrap resampling [51] (*n*_*boot* = 2000) to characterize how much test performance might reasonably vary under repeated sampling. To further assess the statistical significance of the performance difference between EfficientNet-B3 and EfficientNet-B3+CBAM, McNemar’s test [52] with continuity correction was applied to their paired predictions on the test set.

We also computed statistical measures, including chance-level accuracy, Cohen’s effect size (*h*), and post hoc statistical power [53], to quantify the practical significance of classification performance relative to chance.

We performed all statistical analyses and metric evaluations in Python (version 3.11.13) using SciPy (scipy.stats), NumPy (version 1.26.4), and scikit-learn (version 1.2.2). We trained and validated deep learning model components in PyTorch (version 2.6.0+cu124).

### 3.5. Formulas and Statistical Measures

1. Cross-Entropy Loss (Training)

(1) $$L=-\sum_{i=1}^{C}y_{i}log\left({\hat{y}}_{i}\right)$$

where *C* = number of classes (22); *y_i_* = ground-truth label for class *i*; *ŷ_i_* = predicted probability for class *i*.

2. Accuracy

(2) $$Accuracy=\frac{\sum_{i}{TP}_{i}}{\sum_{i}{support}_{i}}$$

where *TP_i_* = true positives for class *i* (samples correctly predicted as class *i*); support_i_ = number of true (actual) samples belonging to class *i*.

3. Precision

(3) $$Precision=\frac{TP}{TP+FP}$$

4. Recall

(4) $$Recall=\frac{TP}{TP+FN}$$

where *TP*, *TN* = true positives, true negatives; *FP*, *FN* = false positives, false negatives.

5. F1-score

(5) $$F_{1}=\frac{2\times Precision\times Recall}{Precision+Recall}$$

6. Matthews Correlation Coefficient (MCC)

(6) $$MCC=\frac{TP\times TN-FP\times FN}{\sqrt{\left(TP+FP\right)\left(TP+FN\right)\left(TN+FP\right)\left(TN+FN\right)}}$$

where *TP*, *TN*, *FP*, and *FN* are as defined above, computed per-class (one-vs.-rest) and then reported individually and as an overall multi-class value.

7. Macro-Averaging (Precision, Recall, F1)

(7) $$Macro-Precision=\frac{1}{C}\sum_{i=1}^{C}{Precision}_{i}$$

The same pattern applies to Macro-Recall and Macro-F1, each computed per-class and then averaged equally across all *C* = 22 classes, independent of class sample size. Note: overall Accuracy is not macro-averaged; it is computed as the proportion of correctly classified samples across the entire test set.

8. Mean Validation Accuracy (Cross-Validation)

(8) $$\bar{A}=\frac{1}{K}\sum_{i=1}^{K}A_{i}$$

where *K* = number of cross-validation folds (K = 3); *A_i_* = validation accuracy obtained in fold i.

9. Standard Deviation

(9) $$SD=\sqrt{\frac{1}{K-1}\sum_{i=1}^{K}{\left(A_{i}-\bar{A}\right)}^{2}}$$

where *K* = number of cross-validation folds (K = 3); *A_i_* = validation accuracy obtained in fold *i*; *Ā* = mean validation accuracy across all folds.

10. 95% Confidence Interval (t-distribution)

(10) $${CI}_{95\%}=\bar{A}\pm t_{K-1,0.975}\frac{SD}{\sqrt{K}}$$

df = *K* − 1 = 2 degrees of freedom, consistent with the three-fold cross-validation design (implemented via scipy.stats.t.interval).

11. Cohen’s h (Effect Size)

(11) $$h=2{sin}^{-1}\left(\sqrt{p_{1}}\right)-2{sin}^{-1}\left(\sqrt{p_{2}}\right)$$

where *p_1_* = observed classification accuracy; *p_2_* = chance-level accuracy (= 1/*C* = 1/22).

## 4. Results

### 4.1. Experimental Setup

All experiments were performed on the Kaggle cloud system (Kaggle Inc., San Francisco, CA, USA) with NVIDIA Tesla P100 and T4 GPUs (NVIDIA Corporation, Santa Clara, CA, USA), each with 16 GB RAM and CUDA-enabled GPU support (CUDA 12.4). We wrote it in Python 3.11.13 (Python Software Foundation, Wilmington, DE, USA) using PyTorch 2.6.0+cu124 (Linux Foundation, San Francisco, CA, USA) and standard libraries, including torchvision 0.21.0+cu124 (Linux Foundation, San Francisco, CA, USA), Pillow (PIL) 11.2.1, NumPy 1.26.4 (NumFOCUS, Austin, TX, USA), and scikit-learn 1.2.2 (Inria, Rocquencourt, France). We kept software versions identical across all experiments to remove variability that could arise from differing library or framework versions.

We preprocessed the input images according to the experimental pipeline used in this study. Training was performed by resizing images to 300 × 300, applying random horizontal flipping (default probability *p* = 0.5) and random rotation (±15°) for data augmentation, and normalizing to a mean of [0.5, 0.5, 0.5] and standard deviation of [0.5, 0.5, 0.5]. We resized and normalized the validation and test images in the same way, without augmentation, to keep the evaluation consistent.

The EfficientNet-B3 backbone was initialized with ImageNet-pretrained weights (EfficientNet_B3_Weights.DEFAULT, torchvision). We applied a fixed random seed (seed = 42) across all random number generators (Python random, NumPy, and PyTorch, including CUDA), and a fixed generator was used for the train/validation split and data loader shuffling, to support reproducibility; minor numerical variation may nonetheless occur across different hardware/CUDA configurations, consistent with standard GPU non-determinism. We did not apply a class-balancing method (e.g., class-weighted loss or oversampling); the model was trained on the natural class distribution of MultiBoneX, which we acknowledge as a contributing factor to reduced performance in minority classes (Section 9).

The experiments in training were carried out in 20 epochs with 32 batch size. We optimized the models via the Adam optimizer [54] with a learning rate of 1 × 10^−4^ and weight decay of 1 × 10^−4^ (L2 regularization) and used categorical cross-entropy loss (1) to perform multi-class classification with 22 classes. Each epoch involved shuffling of the training data to enhance generalization. Training and validation subsets were created, and a random split of 90% and 10% was used on the model development (training) portion of the dataset; the test set was reserved for final evaluation.

A ReduceLROnPlateau learning rate scheduler (factor = 0.5, patience = 2 epochs) reduced the learning rate when validation loss plateaued. We saved model checkpoints whenever validation loss improved and reloaded the checkpoint with the lowest validation loss across all training epochs for final test-set evaluation. We also applied early stopping (patience = 5 epochs), halting training if validation loss failed to improve for 5 consecutive epochs to prevent overfitting.

In addition to the single train/validation/test split, we conducted 3-fold cross-validation on the training data to further verify the stability and generalization of model performance across different data partitions (see Section 3.4 for details).

During training, we monitored validation performance every epoch to help avoid overfitting and inform model selection. We used Kaggle’s standard GPU acceleration and applied no mixed-precision or other GPU-specific optimizations. This setup provided an efficient, repeatable, and reliable way to conduct all experiments, including model training, validation, and testing.

### 4.2. Performance Metrics

We quantitatively assessed model performance using standard statistical metrics to ensure an objective, comprehensive evaluation across both anatomical and fracture categories. During training, we continuously monitored model convergence through training and validation loss and accuracy curves, enabling early detection of overfitting and stable optimization behavior.

For the final evaluation, we measured performance on a 22-class classification task. To provide a balanced, class-agnostic assessment, we macro-averaged Precision, Recall, and F1-score across the 22 output categories to ensure equal contribution regardless of class size, while computing overall Accuracy as the proportion of correctly classified samples across the entire test set.

The four principal performance indicators, Accuracy, Precision, Recall, and F1-score, were computed using the scikit-learn metrics library as (2), (3), (4) and (5), respectively.

These metrics jointly characterize the model’s predictive reliability, class-wise sensitivity, and overall generalization capability, particularly crucial for multi-class radiographic image classification tasks involving overlapping anatomical features.

### 4.3. Training and Validation

We monitored the training behavior of the proposed EfficientNet-B3 + CBAM model based on training and validation accuracy and loss rates. The model was trained for a maximum of 20 epochs using the Adam optimizer with a learning rate of 1 × 10^−4^. We used early stopping with a patience of 5 epochs based on validation loss and retained the best-performing checkpoint for final evaluation. The model converged quickly and steadily in the initial epochs. Training accuracy increased steadily to about 99%, while validation accuracy remained relatively stable between about 87% and 90%. The curves show 8 epochs because early stopping terminated the run before reaching the maximum of 20 epochs. These results reflect a single train/validation/test split. Complementary 3-fold cross-validation was also performed. Across the three folds, the model achieved a mean training accuracy of 98.48% (SD = 0.11%) and a mean validation accuracy of 89.21% (SD = 0.49%), with individual fold validation accuracies of 88.66%, 89.60%, and 89.37%. A 95% confidence interval for the mean validation accuracy was [87.98%, 90.44%], and the close agreement between the single-split validation accuracy and the 3-fold cross-validation results indicates consistent performance across different validation partitions.

The training loss decreased continuously throughout the observed epochs, whereas the validation loss decreased during the initial epochs and subsequently increased. This divergence between training and validation loss suggests substantial overfitting, while the relatively stable validation accuracy indicates the model maintained reasonably consistent classification performance on the validation data.

**Figure 3:**
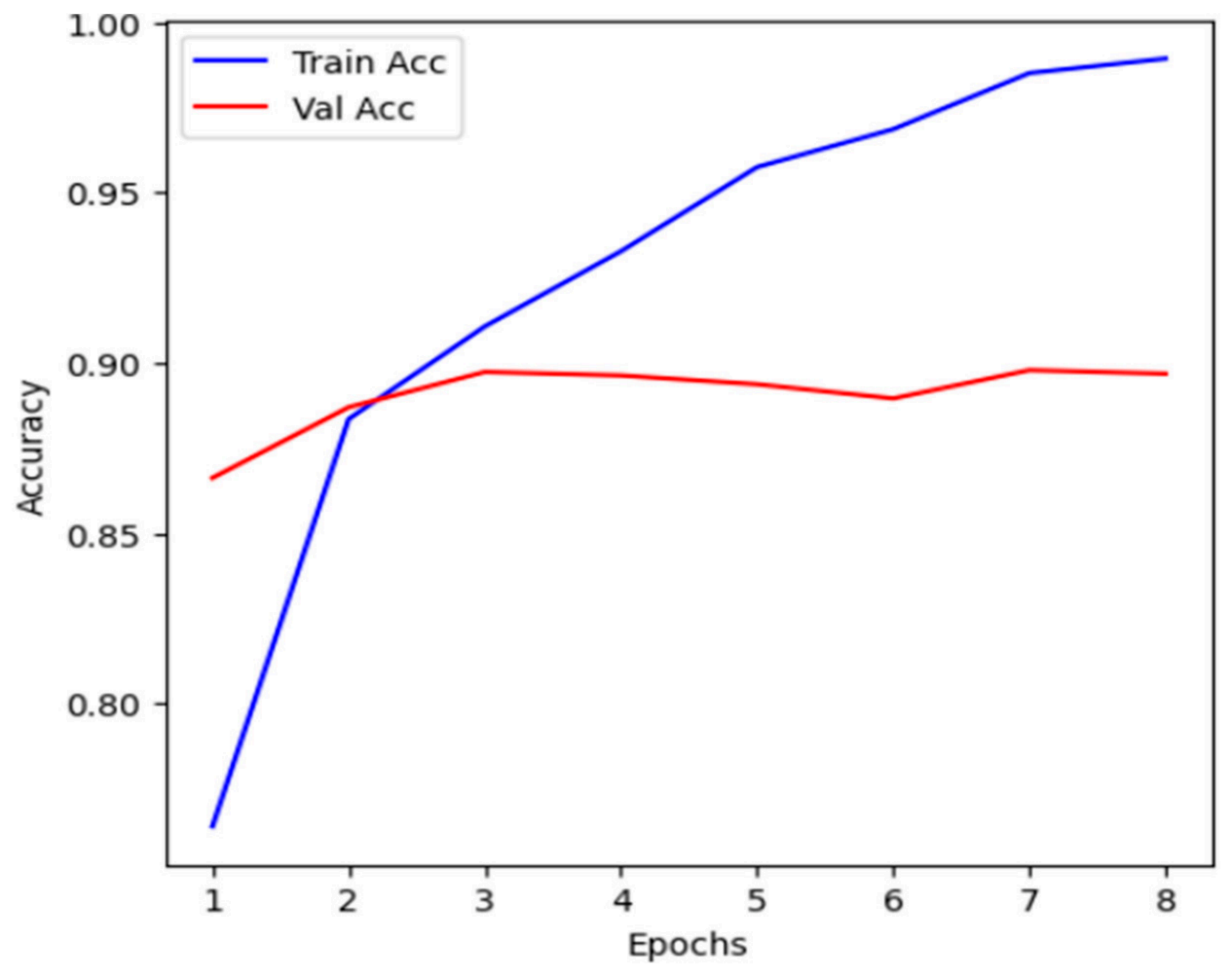
Accuracy Curves.

**Figure 4:**
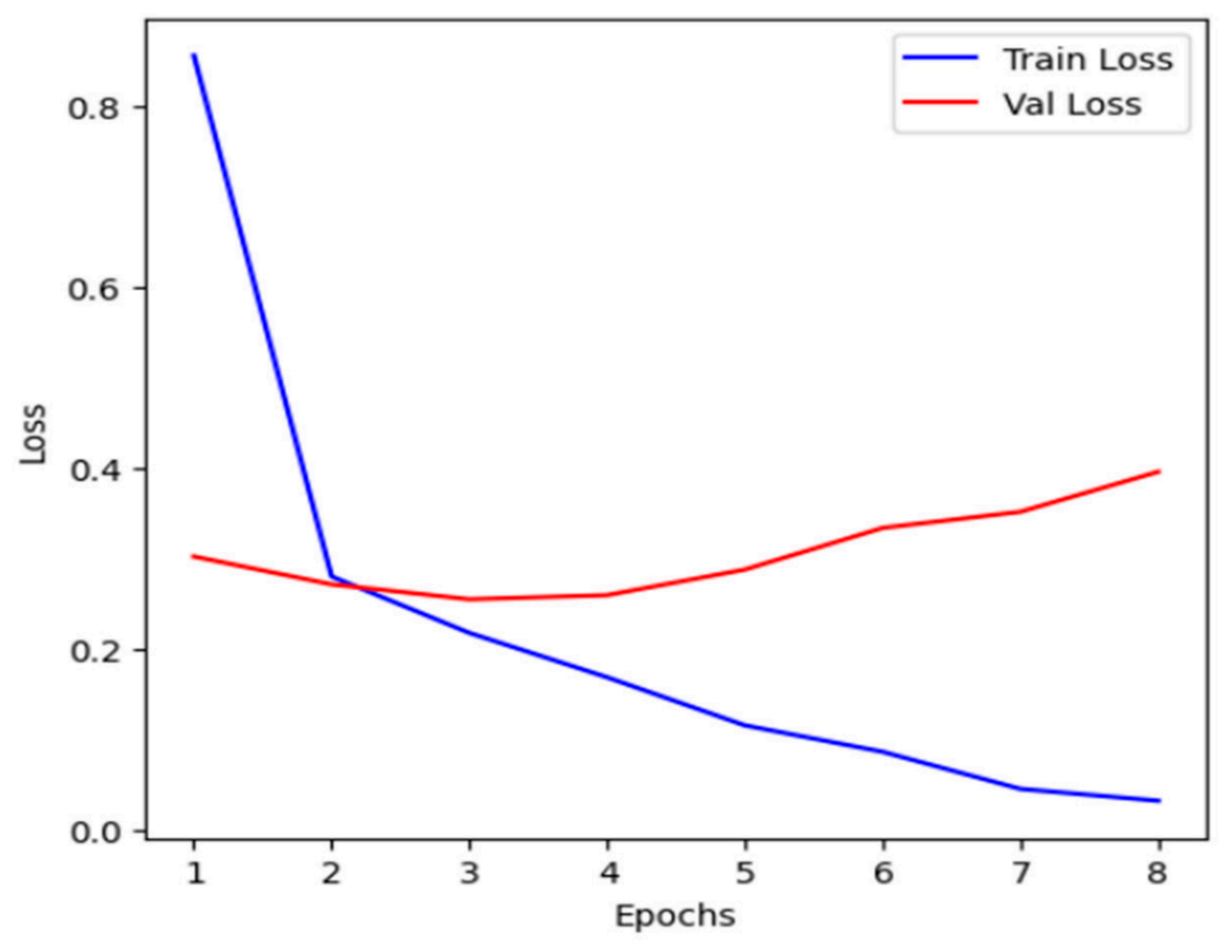
Loss Curves.

Overall, the training and validation results show that EfficientNet-B3 + CBAM learned the multi-class classification task, while also indicating overfitting that should be considered when interpreting its generalization performance. Figure 3 and Figure 4 show the associated accuracy and loss curves, respectively.

### 4.4. Testing and Evaluation

We further assessed the model’s performance on the held-out test set to characterize both the statistical reliability and the qualitative error behavior of the proposed EfficientNet-B3 + CBAM model. Beyond the reported point estimates for accuracy, precision, recall, and F1-score, we computed 95% confidence intervals for the four principal test-set metrics using bootstrap resampling (n_boot = 2000). The resulting intervals were accuracy, 75.03% (95% CI: 73.57–76.43%); macro-precision, 77.09% (95% CI: 75.28–78.77%); macro-recall, 72.85% (95% CI:71.00–74.60%); and macro-F1-score, 73.45% (95% CI: 71.49–75.15%). These narrow, tightly bounded intervals indicate that the reported test scores are stable and unlikely to fluctuate substantially due to random sampling.

As this study uses a fixed, publicly available benchmark dataset rather than prospectively collected data, no a priori sample-size calculation was performed. To assess practical significance, we compared model performance against chance-level accuracy, which for this 22-class problem is 4.55% (1/22). The proposed model achieved an observed test accuracy of 75.03%, corresponding to a large effect size (Cohen’s h ≈ 1.665) relative to chance. The a posteriori statistical power for this comparison was approximately 1.000, and the 95% bootstrap confidence interval for accuracy [0.7357, 0.7643] confirms this estimate is precise given the available test sample size (*n* = 3280). Overall, these results show that our model clearly outperforms random guessing, but predictions for low-support classes like LegANKLE_negative and LegFOOT_positive (*n* = 39) carry more uncertainty due to their small sample size.

The accuracy decreased from training (>99%) to validation (89.21%) and further to the held-out test set (75.03%), following the same pattern as the class distribution across these sets. Training data was approximately balanced (ratio ≈ 1.09:1), validation data had mild imbalance (ratio ≈ 1.7:1), and test data had substantial imbalance (ratio ≈ 6.6:1). This suggests that the growing imbalance across splits may be a major contributor to the accuracy gap. Region- and class-specific difficulty may also play a role: certain regions, such as Shoulder and Wrist, showed lower fracture-detection sensitivity in the test set, likely because fractures are visually subtle in these regions. Differences in image quality and acquisition conditions across the pooled source datasets may also contribute to inconsistent performance for specific classes. Overall, this gap likely reflects a combination of factors—distribution mismatch, region- and class-specific difficulty, image quality variation, and some degree of overfitting to the training distribution—though this study did not separate their individual contributions.

**Figure 5:**
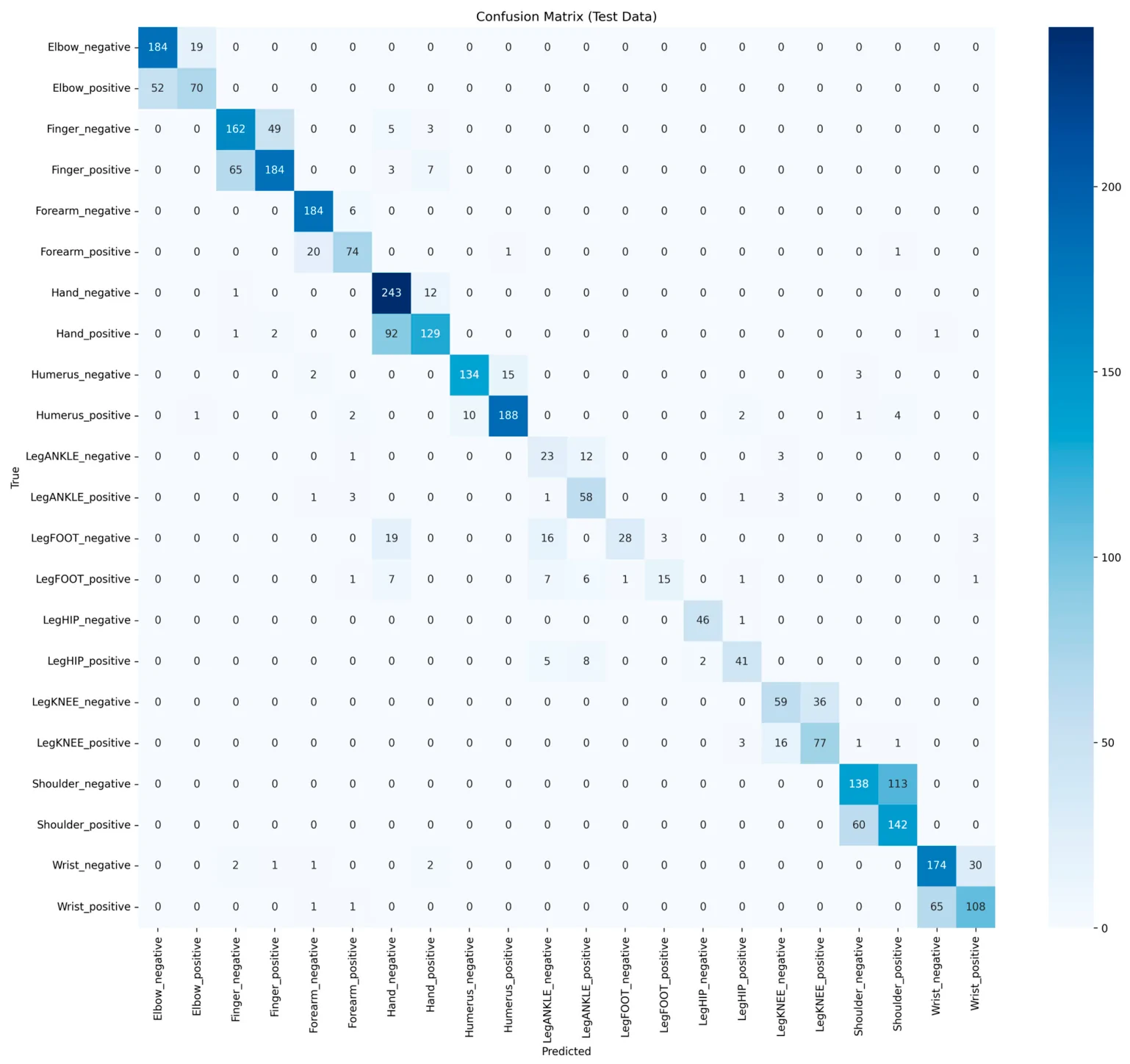
Confusion Matrix of the proposed model on held-out test set.

To complement these quantitative measures, we generated a confusion matrix (Figure 5) to visualize the class-wise prediction distribution and misclassification patterns. The matrix’s diagonal structure shows that the model correctly classified substantial numbers of samples for several anatomical regions, particularly Forearm, Humerus, Elbow, and LegHip, indicating comparatively reliable discrimination among classes in these regions. This pattern is consistent with the stronger region- and class-level performance reported in Section 4.5 and Table A1.

The confusion matrix reveals two distinct error patterns. The first, affecting LegANKLE, LegFOOT, and LegKNEE particularly, involves cross-region misclassification, where samples are assigned to a different anatomical region. For example, several LegFOOT and LegKNEE samples are confused with neighboring lower-limb regions, indicating that distinguishing anatomically adjacent structures remains challenging. Such errors may arise from similarities in anatomical appearance and radiographic projection, although the present study does not directly isolate the underlying causes.

The second pattern involves within-region fracture-status confusion, in which the correct anatomical region is identified but a fractured case is classified as normal. This pattern is particularly evident for Wrist, Shoulder, and Hand. Specifically, 65 of 175 Wrist-positive cases, 60 of 202 Shoulder-positive cases, and 92 of 225 Hand-positive cases were predicted as their corresponding negative classes. These positive-to-negative errors represent false-negative fracture predictions when the task is interpreted as fracture versus no-fracture within a known anatomical region. These errors matter clinically because missed fractures may lead to delayed diagnosis or inappropriate management.

Overall, the confusion matrix shows that the model can learn meaningful anatomical and fracture-status distinctions, while also revealing specific failure modes not fully captured by overall accuracy alone. In particular, the concentration of within-region false negatives in the Wrist, Shoulder, and Hand regions highlights the need to improve fracture sensitivity in these anatomically challenging categories. The subsequent region-wise and class-wise analyses examine the regional and class-level implications of these error patterns.

To further validate model performance under the class imbalance shown in Table 2, we computed the Matthews Correlation Coefficient (MCC) on the test set. MCC remains a reliable indicator even when class sizes are unequal. A one-vs.-rest MCC was computed for each class in addition to the overall multi-class MCC. The following Table 3 shows per-class MCC, along with the overall value.

The overall multi-class MCC of 0.7361 indicates strong agreement between predicted and true labels despite unequal class support. Per-class MCC values ranged from 0.5040 (LegANKLE_negative) to 0.9680 (LegHIP_negative), with lower-support classes such as LegANKLE and LegKNEE showing comparatively variable MCC values, consistent with the confusion matrix patterns discussed above.

### 4.5. Region-Wise Fracture Detection Performance

To support clinically meaningful evaluation within each anatomical region, we also report fracture vs. no-fracture performance separately for each of the 11 anatomical regions. The overall values in Table 4 are obtained by collapsing the 22-class predictions into region-specific binary fracture-status outcomes and therefore differ from the macro-averaged 22-class metrics reported in Table A1. Table 4 shows region-wise performance.

Performance varied considerably across anatomical regions. In terms of sensitivity (fracture detection rate), Humerus performed best (0.9471), followed by LegANKLE (0.9254). At the same time, Elbow (0.5738) and Hand (0.5822) showed the lowest sensitivity, indicating comparatively higher rates of missed fractures in these regions. For specificity, LegHIP achieved the highest value (0.9787), followed by Forearm (0.9684), while LegKNEE (0.6211) was lowest, reflecting a comparatively high false-positive rate. For precision, LegHIP again led (0.9800), followed by Humerus (0.9292), while Shoulder (0.5569) and LegKNEE (0.6923) showed comparatively lower precision. For F1-score, Humerus was highest (0.9381), followed by LegHIP (0.9245) and LegANKLE (0.8732), while Shoulder (0.6214) and Wrist (0.6877) showed the lowest F1-scores. For accuracy, Humerus again led (0.9282), followed by LegHIP (0.9223) and Forearm (0.9091), while Shoulder (0.6181) and LegKNEE (0.7254) showed comparatively lower accuracy.

Overall, Humerus, LegHIP, and Forearm were among the most reliably classified regions across the reported metrics, while Shoulder, Wrist, and Elbow showed comparatively weaker performance across several metrics, particularly sensitivity. LegHIP’s high precision (0.9800) and specificity (0.9787) should be interpreted cautiously given its comparatively small support (*N* = 103) and only one false positive. LegKNEE stood out specifically for its low specificity (0.6211), indicating a tendency toward false positives in that region. Aggregated across all regions, the model achieved an overall sensitivity of 0.7317, specificity of 0.8234, precision of 0.7872, F1-score of 0.7585, and accuracy of 0.7802 for the binary fracture-detection task.

These region-level disparities suggest that fracture-detection difficulty is not uniform across anatomical sites and may relate to factors such as unequal class support within a region, the visual subtlety of fracture patterns in certain bone structures, or differences in image quality and acquisition characteristics across the pooled source datasets. Section 9 (Limitations) discusses these regional performance gaps in more detail.

From a clinical perspective, the observed regional variation is important when considering potential use of the model. The overall test accuracy of 75.03% demonstrates the feasibility of the proposed unified classification framework but does not support autonomous diagnosis. In particular, the lower fracture-detection sensitivity observed for regions such as the Wrist and Shoulder indicates that false-negative predictions remain an important limitation. The model may therefore be better considered as an assistive tool for preliminary triage or decision support, where a qualified clinician reviews predictions, rather than as a standalone diagnostic system. Establishing clinical utility would require external validation, comparison with radiologists, and prospective evaluation under clinically representative conditions.

## 5. Ablation Study

To identify the most suitable backbone for this task, we compared seven pretrained CNN architectures as baselines: EfficientNet-B0, EfficientNet-B3, and EfficientNet-B4 [8], ResNet-18 and ResNet-50 [55], DenseNet-121 [56], and MobileNet-V2 [57]. All models were trained under identical preprocessing, augmentation, and optimization settings (Section 4.1) to ensure a fair comparison.

As shown in Table 5, among the baseline architectures, EfficientNet-B0 demonstrates the best overall performance, achieving the highest accuracy (0.7311), precision (0.7390), and F1-score (0.7017). This indicates strong, well-balanced classification for bone X-ray images, effectively managing the trade-off between false positives and false negatives. Its compound scaling strategy enables efficient extraction of fine-grained bone structures, which is particularly important for medical imaging datasets. EfficientNet-B3 follows closely, showing comparable accuracy (0.7305) and recall (0.7017), suggesting a slightly stronger sensitivity to pathological cases. However, EfficientNet-B0 remains the most robust baseline because it balances all evaluation metrics best.

We augmented the baseline models with Squeeze-and-Excitation (SE) [58] block. As shown in Table 6, among SE-based experiments, EfficientNet-B3 + SE achieves the best performance, with the highest accuracy (0.7332), recall (0.7061), and F1-score (0.7048). The Squeeze-and-Excitation (SE) mechanism enhances channel-wise feature recalibration, allowing the model to emphasize diagnostically relevant bone features more effectively. This improves sensitivity while maintaining stable precision. Although other SE-based models show competitive results, EfficientNet-B3 + SE provides the most consistent and reliable improvement, making it the strongest SE-augmented architecture for bone X-ray classification in this study (SE is used here only as a benchmarking attention mechanism, not as part of the final proposed model).

The Squeeze-and-Excitation (SE) module applies global average pooling followed by a channel reduction (reduction ratio r = 16) via a 1 × 1 convolution, a ReLU activation, a 1 × 1 convolution that restores the original channel dimension, and a sigmoid gate that rescales the input feature map channel-wise.

We then augmented the baseline models with the Convolutional Block Attention Module (CBAM) [9], which extends channel attention with an additional spatial attention mechanism. In Table 7, among the base, SE-augmented, and CBAM-augmented models, the proposed EfficientNet-B3 + CBAM clearly outperforms the others, achieving the highest accuracy (0.7503), precision (0.7709), recall (0.7285), and F1-score (0.7345). This substantial improvement highlights the effectiveness of combining EfficientNet-B3′s compound scaling with CBAM’s joint channel and spatial attention, enabling the model to focus on the most informative regions and structures within bone X-ray images. The strong recall further indicates reduced missed detections, which is critical in clinical settings.

We applied McNemar’s test to the paired predictions of EfficientNet-B3 and EfficientNet-B3 + CBAM across the full test set of 3280 samples. Both models agreed on 2760 samples and disagreed on the remaining 520 samples. Among these samples, EfficientNet-B3 + CBAM correctly classified 294 cases that were misclassified by EfficientNet-B3, whereas EfficientNet-B3 correctly classified only 226 cases that were misclassified by EfficientNet-B3 + CBAM. This difference was statistically significant (χ^2^ = 8.63, *p* = 0.0033 < 0.05, with continuity correction), indicating that the performance improvement from incorporating the CBAM attention mechanism was unlikely to be due to random variation in the test set.

The CBAM module applies sequential channel and spatial attention: the channel attention sub-module uses the same squeeze-excitation structure (global average pooling, 1 × 1 convolution reduction with r = 16, ReLU, 1 × 1 convolution expansion, sigmoid) to rescale channels, followed by a spatial attention sub-module that concatenates channel-wise average- and max-pooled feature maps and applies a 7 × 7 convolution with a sigmoid activation to produce a spatial attention map.

Overall, EfficientNet-B3 + CBAM represents the most powerful and reliable model across all experimental configurations, confirming it as the proposed CBAM-augmented architecture adopted for this study.

### Computational Comparison and Backbone Selection Justification

We used EfficientNet-B3 as the baseline architecture for computational comparison and selected EfficientNet-B3 + CBAM and EfficientNet-B4 + CBAM as the two best-performing models among all evaluated architectures. We then compared their computational characteristics with the B3 baseline to assess the efficiency trade-offs of the proposed attention-based architecture and the larger B4 backbone. For a fair computational comparison, we evaluated all three models under a fixed training budget of 10 epochs, with early stopping disabled specifically for this analysis. We adopted this fixed-epoch protocol because early stopping could cause models to complete different numbers of epochs, making total training times difficult to compare fairly. Therefore, we trained all three models for exactly 10 epochs under the same evaluation protocol. We then compared training time, model complexity, and inference efficiency across the three architectures.

EfficientNet-B3 contained 10,730,046 trainable parameters and required 3.860 GFLOPs per image. Under the fixed 10-epoch evaluation, its average training time was 332.62 s per epoch, resulting in a total training time of approximately 55.44 min. The measured single-image inference time was 13.7928 ms.

EfficientNet-B4 + CBAM contained 17,989,568 trainable parameters and required 5.998 GFLOPs per image. Under the fixed 10-epoch protocol, its average training time was 637.84 s per epoch, or about 106.31 min. Its measured single-image inference time was 19.4332 ms.

EfficientNet-B3 + CBAM contained 11,025,056 trainable parameters and required 3.861 GFLOPs per image. Its average training time was 333.40 s per epoch, resulting in a total training time of approximately 55.57 min. The measured single-image inference time was 13.6300 ms. Table 8 summarizes these computational comparisons.

Based on the computational results and the predictive performance on the test set, we selected EfficientNet-B3 + CBAM as the proposed architecture. Compared with the EfficientNet-B3 baseline, it required only 295,010 additional trainable parameters and 0.001 higher GFLOPs, while achieving a 0.163 ms faster single-image inference time. The average training-time difference was also marginal, with B3 + CBAM requiring only 0.78 s more per epoch. These differences indicate that the addition of CBAM introduced minimal computational overhead while maintaining a comparable computational profile and slightly improving single-image inference efficiency. In contrast, EfficientNet-B4 + CBAM imposed substantially higher computational and inference costs. Most importantly, EfficientNet-B3 + CBAM achieved the best overall test-set predictive performance among the evaluated models. Thus, its combination of superior predictive performance, low inference latency, and computational requirements close to the B3 baseline motivated its selection as the final proposed model.

## 6. Grad-CAM Analysis

To improve the interpretability of the proposed model (EfficientNet-B3 + CBAM), we employed Grad-CAM to visualize the image regions most strongly associated with the model’s predictions. We applied Grad-CAM to selected samples using the same preprocessing and normalization strategy used during validation and testing, without data augmentation, to ensure consistency between the quantitative evaluation and the visual explainability analysis. We verified each image’s ground-truth region and fracture-status label against the dataset class from which it was drawn; however, we did not independently annotate or have a radiologist confirm precise pixel-level fracture location, so we describe Grad-CAM activation qualitatively rather than validating it as spatially accurate fracture localization.

**Figure 6:**
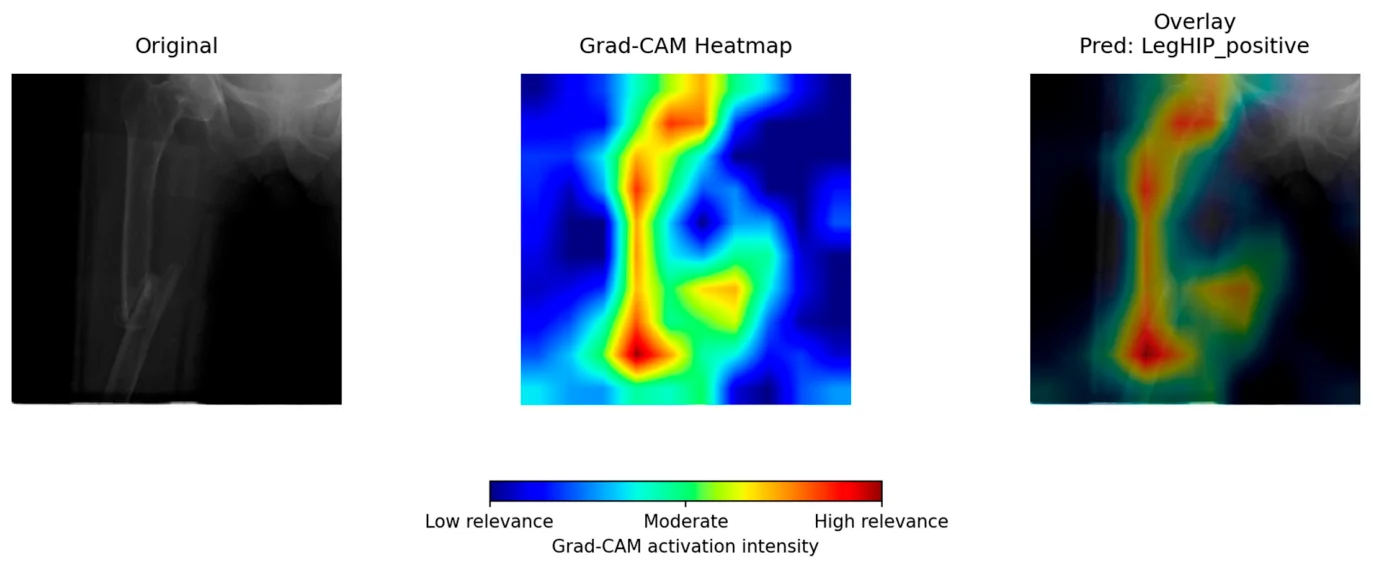
Grad-CAM visualization of a correctly classified LegHIP_positive case.

**Figure 7:**
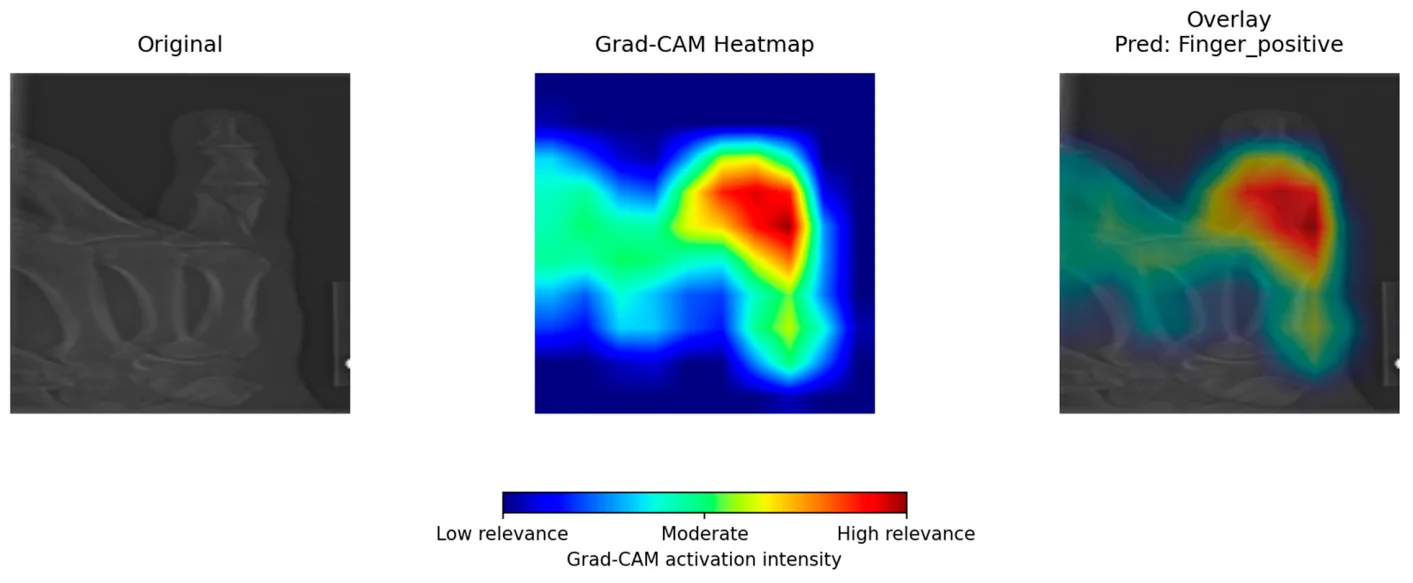
Grad-CAM visualization of a correctly classified Finger_positive case.

**Figure 8:**
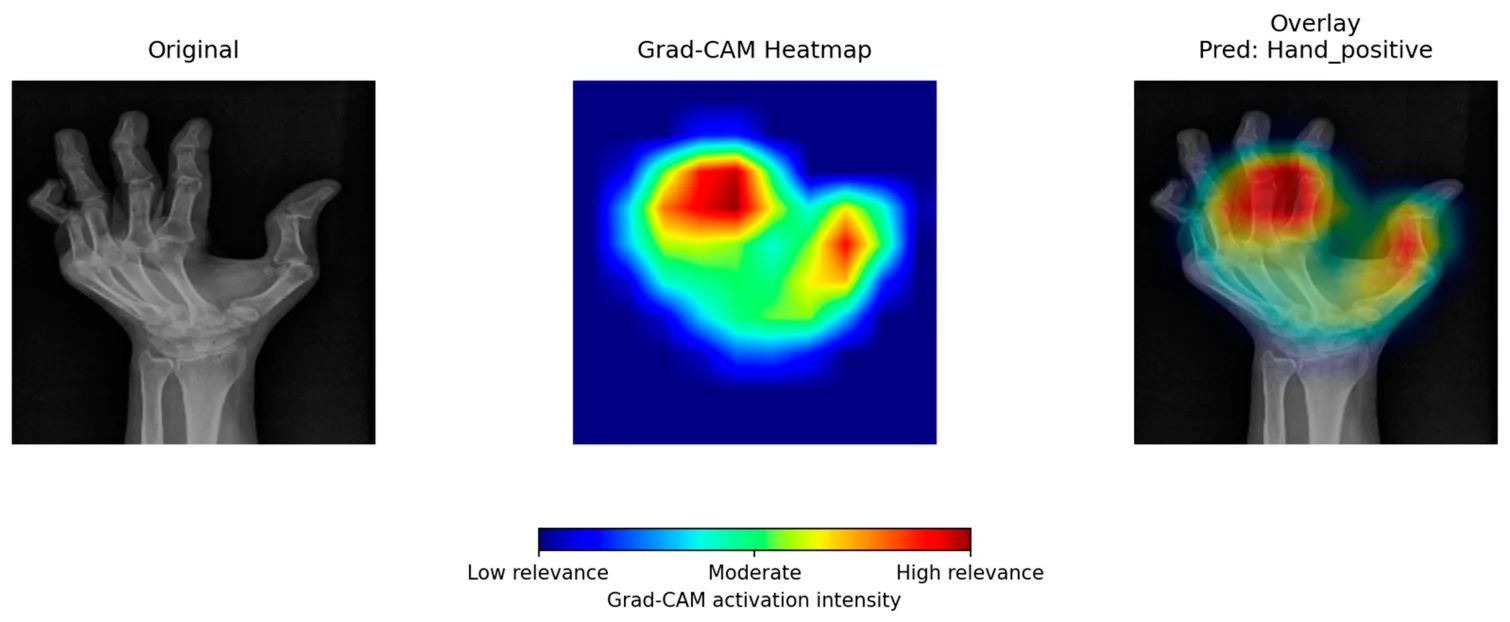
Grad-CAM visualization of a correctly classified Hand_positive case.

**Figure 9:**
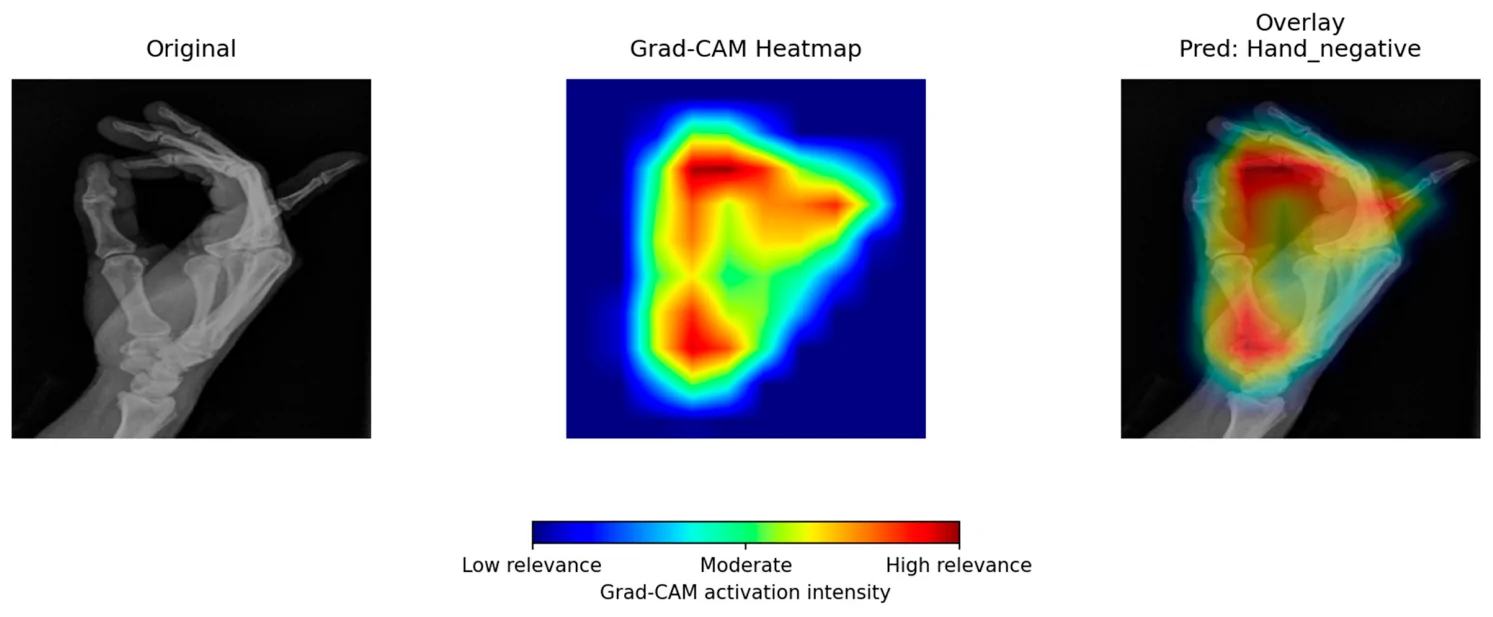
Grad-CAM visualization of a correctly classified Hand_negative case.

**Figure 10:**
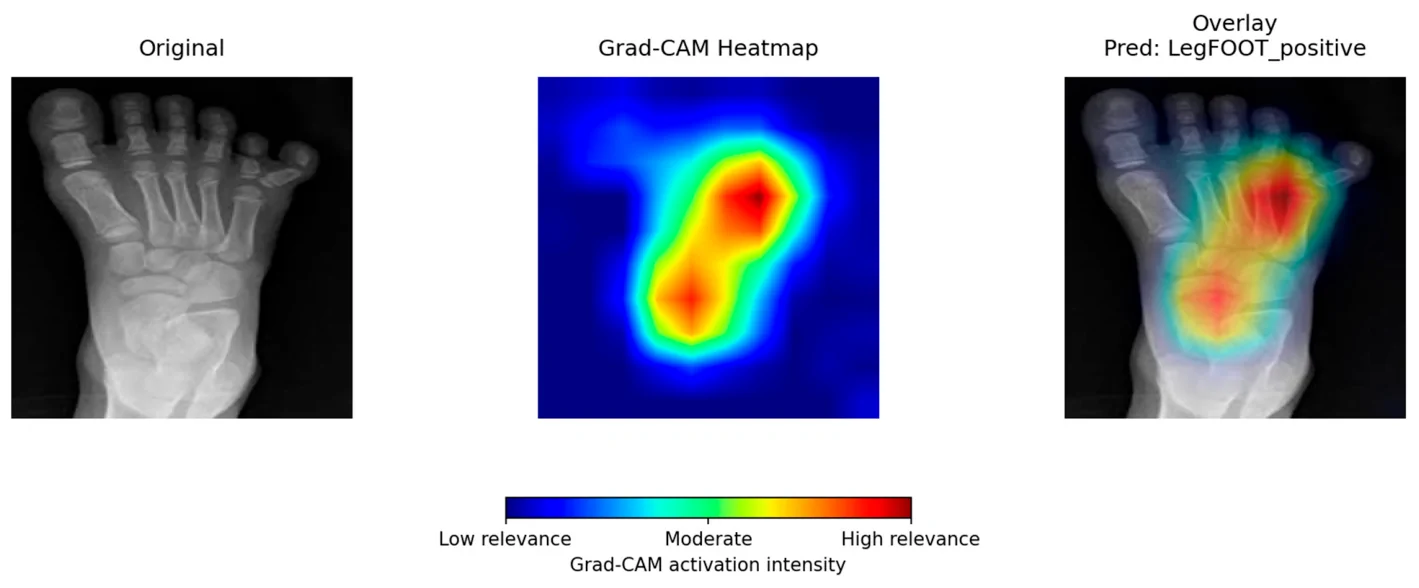
Grad-CAM visualization of a correctly classified LegFOOT_positive case.

**Figure 11:**
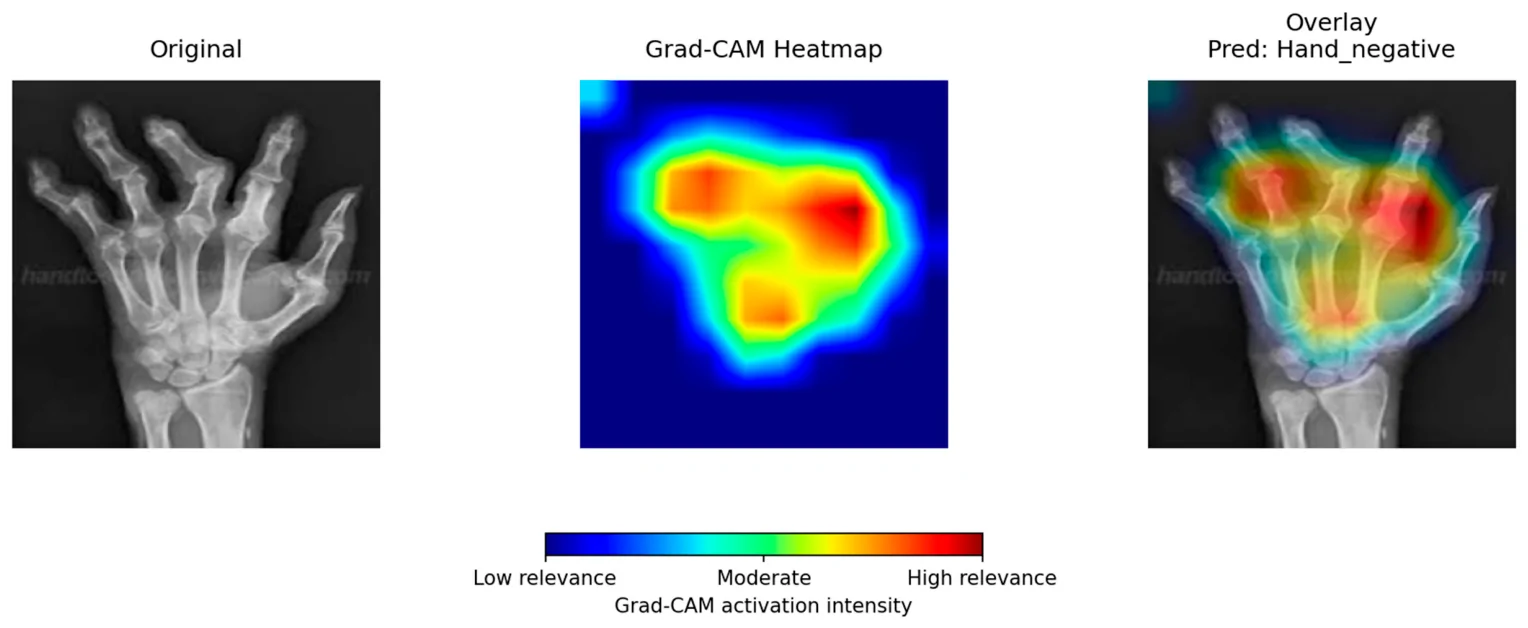
Grad-CAM visualization of a misclassified Hand_positive case as Hand_negative.

We selected a few representative test-set images to illustrate the model’s behavior across the range of outcomes observed in this study. Figure 6, Figure 7 and Figure 8 depict cases in which the model correctly predicted the positive (fracture-present) class, consistent with the dataset’s annotated label for each image. Figure 9 shows a case correctly predicted as negative (no fracture present), consistent with the corresponding annotated label. Figure 10 again shows a correctly classified positive case. Figure 11, by contrast, illustrates a misclassification, in which the model predicted the negative class despite the annotated label indicating a fracture was present. Together, these six examples span the three prediction outcomes relevant to this task: correct positive classification, correct negative classification, and misclassification.

To complement these qualitative visualizations, we performed a quantitative Grad-CAM analysis on a balanced subset of 220 test images, comprising 10 images from each of the 22 classes. For each image, we calculated the fraction of the image exhibiting high Grad-CAM activation, mean activation, and peak activation. Peak activation values remained consistently high across the evaluated classes, ranging from 0.9904 for LegFOOT_negative to 0.9966 for Humerus_negative. In contrast, the spatial coverage and mean activation varied more substantially across classes. LegHIP_positive showed the highest high-activation coverage (0.3716) and mean activation (0.3531), whereas LegFOOT_negative showed the lowest high-activation coverage (0.0732). Hand_negative (0.2079) and Humerus_negative (0.1709) also showed relatively high activation coverage. These results indicate that the spatial extent and average strength of model activation varied across anatomical regions and fracture-status categories, while strongly activated regions were present across all evaluated classes.

Overall, the qualitative and quantitative Grad-CAM analyses provide complementary insights into the model’s prediction behavior. The visualizations illustrate representative attention patterns and failure cases, while the quantitative analysis characterizes the extent and strength of activation across classes. As noted above, these findings should be interpreted as evidence of model attention patterns rather than validated fracture localization.

## 7. Comparison with Existing Work

Several studies have investigated fracture detection and classification using machine learning and deep learning approaches, reporting different accuracy levels depending on dataset type and classification complexity. Researchers initially applied traditional machine learning methods to binary fracture classification problems, such as an SVM-based approach by Ahmed et al. [21], which reported 92.86% accuracy on private data. With advances in deep learning, Lee et al. [38] used an Inception-ResNet-V2 model integrated with attention U-Net segmentation for a four-class classification task, achieving accuracy ranging from 69% to 88% across cross-validation folds. Krogue et al. [33] used a DenseNet architecture for six-class fracture classification and achieved 90.8% accuracy on a private dataset. Cheng et al. [59] proposed a transfer-learning-based DCNN pipeline for binary fracture classification and reported 91% accuracy on private data. Also, El-Saadawy et al. [28] proposed a MobileNet-based approach and achieved an average accuracy of 73.42% in upper-body bone X-ray abnormality detection.

Researchers have also explored attention mechanisms and hybrid architectures to improve performance. Bae et al. [60] used a ResNet-18 model with a CBAM attention module for a three-class classification task, achieving 98.6% accuracy on internal data and 97.1% on external data. Chen et al. [61] investigated CNN and ResNeXt architectures for binary fracture classification and reported 73.59% accuracy using a private dataset. Studies using public datasets are comparatively limited; however, Abd El-Ghany et al. [62] reported 99.60% accuracy for binary classification using MLFNet, while Barhoom et al. [42] achieved 85.96% precision across 14 classes using a VGG16-based CNN.

Our EfficientNet-B3+CBAM model addresses a 22-class joint region-and-fracture-status classification task on a public dataset and achieves 75.03% accuracy (95% CI: 73.57-76.43%). To the best of our knowledge, no prior published study has addressed a directly comparable 22-class task on a public dataset. Table 9 therefore summarizes the most relevant available studies in fracture classification and detection using radiographic imaging, spanning binary to 14-class formulations; these comparisons should be interpreted as contextual rather than as evidence of superiority or clinical robustness, given substantial differences across studies in number of classes, dataset accessibility (private vs. public), task endpoints, and validation design. Most prior work addresses simpler 2–6 class binary or few-class endpoints on private datasets, which are not directly comparable to the 22-class, public-dataset, and joint region-and-status classification task addressed here. Our performance is broadly consistent with the few prior studies using public, multi-class datasets of comparable complexity (e.g., 73.42% [28]).

## 8. Conclusions

This paper presented a unified deep learning model for multi-region, multi-class bone fracture classification using the publicly available MultiBoneX dataset. The proposed EfficientNet-B3 model with CBAM demonstrated that anatomical-region identification and fracture-status detection can be handled jointly in a single end-to-end system, framing the task as a 22-class classification problem across 11 anatomical regions. On the held-out test set of 3280 images, the model achieved a test accuracy of 75.03% (95% CI: 73.57–76.43%), with precision, recall, and F1-score of 77.09% (95% CI: 75.28–78.77%), 72.85% (95% CI: 71.00–74.60%), and 73.45% (95% CI: 71.49–75.15%), respectively. Additionally, 3-fold cross-validation confirmed that these findings were consistent across different data splits, achieving an average validation accuracy of 89.21% (95% CI: 87.98–90.44%).

The results indicated that adding CBAM improved the model’s ability to focus on subtle fracture-relevant features, facilitating discrimination in complex or visually obstructed body regions. In addition, the MultiBoneX dataset, which incorporates several radiographic repositories, enabled a broad evaluation of multi-region fracture classification models.

Class-wise analysis (Appendix A, Table A1) further showed that this overall performance was not uniform across the 22 categories. Classes such as Forearm_negative, LegHIP_negative, and Humerus_positive achieved F1-scores above 0.89, reflecting strong discrimination where fracture features are visually distinct and well-represented. In contrast, classes such as LegANKLE_negative, LegKNEE_negative, and Shoulder_positive showed markedly lower F1-scores, which may be attributable to limited sample support or anatomical overlap with morphologically adjacent regions, as well as the class-distribution differences between training and evaluation splits discussed in Section 4.4. This variation across classes indicates that overall accuracy alone does not fully capture per-class difficulty. It highlights which anatomical regions are harder to classify and where future work needs additional training data and improved class balancing.

Overall, the results—supported by test-set confidence intervals, cross-validated stability, and class-level diagnostics—indicate that the proposed framework provides a unified and scalable approach to multi-region fracture classification, extending beyond earlier binary or region-specific approaches by jointly classifying 11 anatomical regions with fracture status. The consistency between single-split and cross-validated performance, together with the identified class-level differences, provides a clearer picture of where the model performs reliably and where further refinement is needed.

From a clinical perspective, these results should be interpreted as evidence of feasibility rather than clinical readiness. The model showed relatively strong fracture discrimination for regions such as the Humerus and Forearm, while fracture-detection sensitivity was substantially lower for the Wrist, Shoulder, and LegFOOT regions. Because false-negative fracture predictions may result in missed or delayed treatment, the current model should not be considered suitable for autonomous diagnosis. Its potential role is better characterized as an assistive tool for preliminary triage or decision support, subject to confirmation by a qualified clinician. However, the present evaluation was limited to internal testing without reader comparison or external validation (Section 9). Therefore, external validation, reader-comparison studies, and prospective clinical evaluation are required before considering the model for routine clinical use.

## 9. Limitations

**Patient-level split not verifiable**: MultiBoneX ver1 is a public, de-identified dataset compiled from MURA [46], FracAtlas [47], HBFMID [48], and LERA [49], and it does not provide patient IDs. As a result, we could not confirm whether the same patient appears in more than one split (train/validation/test), so we cannot rule out some patient overlap across splits. 

**Limited samples for some classes**: Limited samples for some classes in validation/test: Classes such as LegANKLE and LegFOOT have relatively few images in the validation and test splits (Table 2). Because we did not stratify the validation/test split by class, this imbalance was not evenly distributed across subsets. As a result, individual results for these classes (Table 3 and Table A1) are less stable and more sensitive to small changes, and this train/test distribution mismatch is also a contributing factor to the training-test accuracy gap.

**Single dataset, no outside testing**: The model was only tested on this one public dataset. It has not yet been tested on images from other hospitals, scanners, or patient groups.

**Region-specific models not evaluated**: The model was trained and tested only as a single 22-class classifier that predicts both anatomical region and fracture status from the image alone. We did not build or test an alternative version where the region is already known in advance and the model only has to decide fracture vs. no fracture. Such a region-specific model may achieve higher fracture-detection accuracy than the joint 22-class model used in this study, but we did not perform this comparison.

**Cross-split duplicate/near-duplicate leakage:** A perceptual-hashing analysis found that 2.81% of the dataset had an identical perceptual hash to an image in a different split, with an additional 711 near-duplicate cross-split pairs. Because the source datasets do not include patient identifiers, we cannot rule out additional leakage from the same patient appearing across splits. Although we identified these duplications through perceptual-hash analysis, we retained the original dataset structure for benchmark comparability. Consequently, the reported test performance may not fully represent patient-independent generalization.

## 10. Future Work

Several directions can be explored to enhance the model’s clinical utility and move it closer to real-world readiness. One potential extension is the integration of localization mechanisms such as bounding-box detectors or attention-guided localization to highlight exact fracture regions; validating such outputs including the current Grad-CAM visualizations against radiologist-confirmed annotations would improve interpretability and clinical trust. Additionally, incorporating multi-modal imaging data, including CT or MRI scans, could strengthen the model’s ability to identify subtle or complex fractures that may not be clearly visible on plain X-rays.

Another important direction is classifying fracture subtypes, such as displaced, comminuted, or intra-articular fractures, which are clinically relevant for treatment planning. Adding subtype-level annotations would allow the model to support more detailed and actionable clinical insights. Furthermore, expanding the dataset to include more diverse demographic groups, imaging protocols, and pathological conditions could increase generalizability across hospitals and patient populations.

Finally, real-world validation through prospective clinical trials, radiologist–AI comparison studies, or integration into PACS/RIS systems will be essential to assess the model’s operational reliability. Stronger regularization should also be explored to further reduce the observed training-test performance gap. Such validation would support its transition from a research prototype to a deployable clinical decision-support tool that can assist practitioners in high-volume radiology environments.

## Figures and Tables

**Table 1:** Anatomical Class Distribution in MultiBoneX ver1 Dataset.

No.	Anatomical Region	Fractured	Normal	Total Images
1	Humerus	1084	1025	2109
2	Elbow	1009	1091	2100
3	Forearm	969	1075	2044
4	Wrist	1056	1098	2154
5	Hand	1113	1144	2257
6	Finger	1147	1106	2253
7	LegKNEE	974	947	1921
8	Shoulder	1086	1139	2225
9	LegANKLE	936	898	1834
10	LegFOOT	906	933	1839
11	LegHIP	915	907	1822
Total	-	11,195	11,363	22,558

**Table 2:** Per-class distribution of images across training, validation, and test sets.

Class Name	Train Count	Val Count	Test Count	Total
Elbow_negative	783	105	203	1091
Elbow_positive	814	73	122	1009
Finger_negative	805	82	219	1106
Finger_positive	811	77	259	1147
Forearm_negative	791	94	190	1075
Forearm_positive	785	88	96	969
Hand_negative	786	102	256	1144
Hand_positive	807	81	225	1113
Humerus_negative	773	98	154	1025
Humerus_positive	780	96	208	1084
LegANKLE_negative	770	89	39	898
LegANKLE_positive	784	85	67	936
LegFOOT_negative	773	91	69	933
LegFOOT_positive	776	91	39	906
LegHIP_negative	769	91	47	907
LegHIP_positive	769	90	56	915
LegKNEE_negative	756	96	95	947
LegKNEE_positive	785	91	98	974
Shoulder_negative	796	92	251	1139
Shoulder_positive	822	62	202	1086
Wrist_negative	802	86	210	1098
Wrist_positive	813	68	175	1056
Total calculated	17,350	1928	3280	22,558

**Table 3:** Per-class MCC.

Class	Support	MCC
Elbow_negative	203	0.8294
Elbow_positive	122	0.6573
Finger_negative	219	0.6997
Finger_positive	259	0.7235
Forearm_negative	190	0.9185
Forearm_positive	96	0.7995
Hand_negative	256	0.7705
Hand_positive	225	0.6778
Humerus_negative	154	0.8951
Humerus_positive	208	0.9068
LegANKLE_negative	39	0.5040
LegANKLE_positive	67	0.7679
LegFOOT_negative	69	0.6216
LegFOOT_positive	39	0.5630
LegHIP_negative	47	0.9680
LegHIP_positive	56	0.7792
LegKNEE_negative	95	0.6636
LegKNEE_positive	98	0.7229
Shoulder_negative	251	0.5829
Shoulder_positive	202	0.5901
Wrist_negative	210	0.7587
Wrist_positive	175	0.6694
Overall (multi-class MCC)	3280	0.7361

**Table 4:** Region-wise Fracture Detection Performance.

Region	N	TP	FN	TN	FP	Sensitivity	Specificity	Precision	F1	Accuracy
Elbow	325	70	52	184	19	0.5738	0.9064	0.7865	0.6635	0.7815
Finger	478	191	68	167	52	0.7375	0.7626	0.7860	0.7610	0.7490
Forearm	286	76	20	184	6	0.7917	0.9684	0.9268	0.8539	0.9091
Hand	481	131	94	244	12	0.5822	0.9531	0.9161	0.7120	0.7796
Humerus	362	197	11	139	15	0.9471	0.9026	0.9292	0.9381	0.9282
LegANKLE	106	62	5	26	13	0.9254	0.6667	0.8267	0.8732	0.8302
LegFOOT	108	24	15	63	6	0.6154	0.9130	0.8000	0.6957	0.8056
LegHIP	103	49	7	46	1	0.8750	0.9787	0.9800	0.9245	0.9223
LegKNEE	193	81	17	59	36	0.8265	0.6211	0.6923	0.7535	0.7254
Shoulder	453	142	60	138	113	0.7030	0.5498	0.5569	0.6214	0.6181
Wrist	385	109	66	177	33	0.6229	0.8429	0.7676	0.6877	0.7429
Overall	3280	1132	415	1427	306	0.7317	0.8234	0.7872	0.7585	0.7802

**Table 5:** Base Models.

Model	Accuracy	Precision	Recall	F1-Score
EfficientNet-B0	0.7311	0.7390	0.7006	0.7017
ResNet-18	0.7015	0.7152	0.6659	0.6687
ResNet-50	0.7137	0.7357	0.7029	0.6963
EfficientNet-B4	0.7040	0.7187	0.6709	0.6671
MobileNet-V2	0.7131	0.7222	0.6836	0.6831
DenseNet-121	0.7137	0.7269	0.6895	0.6914
EfficientNet-B3	0.7305	0.7299	0.7017	0.6992

**Table 6:** Base Models with SE.

Model	Accuracy	Precision	Recall	F1-Score
EfficientNet-B0 + SE	0.7226	0.7470	0.7040	0.7013
ResNet-18 + SE	0.6140	0.6308	0.5786	0.5749
DenseNet-121 + SE	0.7226	0.7348	0.7013	0.6996
ResNet-50 + SE	0.6988	0.7163	0.6679	0.6711
EfficientNet-B4 + SE	0.7232	0.7399	0.7017	0.7028
MobileNet-V2 + SE	0.7040	0.7138	0.6806	0.6821
EfficientNet-B3 + SE	0.7332	0.7276	0.7061	0.7048

**Table 7:** Base Models with CBAM.

Model	Accuracy	Precision	Recall	F1-Score
ResNet-18 + CBAM	0.5762	0.5813	0.5327	0.5352
DenseNet-121 + CBAM	0.7140	0.7475	0.6962	0.7012
ResNet-50 + CBAM	0.6695	0.6766	0.6207	0.6232
MobileNet-V2 + CBAM	0.7222	0.7356	0.7025	0.7065
EfficientNet-B4 + CBAM	0.7335	0.7349	0.6949	0.6947
EfficientNet-B0 + CBAM	0.7235	0.7403	0.6961	0.6984
EfficientNet-B3 + CBAM	0.7503	0.7709	0.7285	0.7345

**Table 8:** Computational comparison of the evaluated architectures.

Model	Trainable Parameters	GFLOPs	Avg. Training Time/Epoch (s)	Total 10-Epoch Time (min)	Inference Time/Image (ms)
EfficientNet-B3	10,730,046	3.860	332.62	55.44	13.7928
EfficientNet-B4 + CBAM	17,989,568	5.998	637.84	106.31	19.4332
EfficientNet-B3 + CBAM	11,025,056	3.861	333.40	55.57	13.6300

**Table 9:** Comparison of the proposed model with existing work.

References	Method (Model)	Classes	Dataset	Accuracy
Ahmed et al. [21]	SVM	2	Private	92.86%
Lee et al. [38]	Inception-ResNet-V2 (with attention U-Net segmentation)	4	Private	69–88%(range across cross-validation folds)
Krogue et al. [33]	DenseNet-169	6	Private	90.8%
El-Saadawy et al. [28]	Two-stage MobileNet-based approach	Stage 1 = 7 Stage 2 = 2	Public	73.42%
Cheng et al. [59]	DCNN(Transfer- learning pipeline)	2	Private	91%
Bae et al. [60]	ResNet-18 + CBAM	3	Private	98.6%(internal); 97.1%(external)
Chen et al. [61]	CNN/ResNeXt	2	Private	73.59%
Abd El-Ghany et al. [62]	MLFNet	2	Public	99.60%
Barhoom et al. [42]	VGG16 CNN	7 × 2 = 14	Public	85.96% precision
Proposed Model	EfficientNet-B3 + CBAM	22	Public	75.03%

## Data Availability

Dataset: https://figshare.com/articles/dataset/MultiBoneX_zip/29936561?file=57269345 (accessed on 27 September 2025).

## Abbreviations

The following abbreviations are used in this manuscript:

CBAM	Convolutional Block Attention Module
CAD	Conventional computer-aided diagnosis
AI	Artificial intelligence
CNNs	Convolutional Neural Networks
ML	Machine Learning
GLCM	Gray-Level Co-occurrence
LBP	Local Binary Patterns
HOG	Histogram of Oriented Gradients
DT	Decision Trees
RF	Random Forests
ROI	Region of Interest
PCA	Principal Component Analysis
RFE	Recursive Feature Elimination
SVM	Support Vector Machine
KNN	k-Nearest Neighbors
AUC	Area Under the Curve
NCGNN	Node-Level Capsule Graph Neural Network
GOA	Gazelle Optimization Algorithm
MURA	Musculoskeletal Radiographs
ROC	Receiver Operating Characteristic
ShPCNNet	Shuffle Parallel Convolutional Neural Network
GPU	Graphics Processing Unit
TP	True Positive
TN	True Negative
FP	False Positive
FN	False Negative
SE	Squeeze-and-Excitation
Grad-CAM	Gradient-weighted Class Activation Mapping
CT	Computed Tomography
MRI	Magnetic Resonance Imaging
PACS	Picture Archiving and Communication System
RIS	Radiology Information System
MCC	Matthews Correlation Coefficient
GFLOPs	Giga Floating-Point Operations per Second
S	Second
Min	Minute
Ms	Millisecond
Avg	Average

## Appendix A. Per-Class Classification Performance

Table A1 provides a detailed breakdown of precision, recall, and F1-score for each of the 22 target classes, along with the corresponding number of test samples (support). This class-wise analysis identifies categories where the model performs reliably versus those needing further optimization, offering a more granular view than aggregate accuracy alone.

**Table A1:** Per-class Classification Report.

Class	Precision	Recall	F1-Score	Support
Elbow_negative	0.7797	0.9064	0.8383	203.0000
Elbow_positive	0.7778	0.5738	0.6604	122.0000
Finger_negative	0.7013	0.7397	0.7200	219.0000
Finger_positive	0.7797	0.7104	0.7434	259.0000
Forearm_negative	0.8804	0.9684	0.9223	190.0000
Forearm_positive	0.8409	0.7708	0.8043	96.0000
Hand_negative	0.6585	0.9492	0.7776	256.0000
Hand_positive	0.8431	0.5733	0.6825	225.0000
Humerus_negative	0.9306	0.8701	0.8993	154.0000
Humerus_positive	0.9216	0.9038	0.9126	208.0000
LegANKLE_negative	0.4423	0.5897	0.5055	39.0000
LegANKLE_positive	0.6905	0.8657	0.7682	67.0000
LegFOOT_negative	0.9655	0.4058	0.5714	69.0000
LegFOOT_positive	0.8333	0.3846	0.5263	39.0000
LegHIP_negative	0.9583	0.9787	0.9684	47.0000
LegHIP_positive	0.8367	0.7321	0.7810	56.0000
LegKNEE_negative	0.7284	0.6211	0.6705	95.0000
LegKNEE_positive	0.6814	0.7857	0.7299	98.0000
Shoulder_negative	0.6798	0.5498	0.6079	251.0000
Shoulder_positive	0.5441	0.7030	0.6134	202.0000
Wrist_negative	0.7250	0.8286	0.7733	210.0000
Wrist_positive	0.7606	0.6171	0.6814	175.0000
accuracy	-	-	-	0.7503
macro average	0.7709	0.7285	0.7345	3280
weighted average	0.7641	0.7503	0.7464	3280

As shown in Table A1, several classes—including Forearm_negative, LegHIP_negative, and Humerus_positive—achieved F1-scores above 0.89, indicating strong and consistent classification performance. In contrast, classes such as LegANKLE_negative, LegKNEE_negative, and Shoulder_positive showed comparatively lower F1-scores, which may be influenced by limited sample support, anatomical characteristics, or overlap with morphologically adjacent regions.
